# Structural and optical properties of the Ag_*n*_–tyrosine complexes (*n* = 3−12): a density functional theory study

**DOI:** 10.1098/rsos.230908

**Published:** 2023-12-13

**Authors:** José Luis Balan, José Aminadat Morato-Márquez, José Gilberto Torres-Torres, José Luis Cabellos, Filiberto Ortiz-Chi

**Affiliations:** ^1^ División Académica de Ciencias Básicas, Universidad Juárez Autónoma de Tabasco, Cunduacán 86690, Tabasco, México; ^2^ CONAHCYT-División Académica de Ciencias Básicas, Universidad Juárez Autónoma de Tabasco, Cunduacán 86690, Tabasco, México; ^3^ Tecnológico Nacional de México, Instituto Tecnológico de Villahermosa, Departamento de Ciencias de la Tierra, Villahermosa, 86010, Tabasco, México; ^4^ Universidad Politécnica de Tapachula, Coordinación de Investigación y Desarrollo Tecnológico, Tapachula 30830, Chiapas, México

**Keywords:** silver capped clusters, optical properties, DFT calculations

## Abstract

We study the optical properties of Ag_*n*_ (*n* = 3–12) neutral clusters and their coordination with a tyrosine (Tyr) molecule. A global search strategy coupled with density functional theory (DFT) computations explored the potential energy surface. Adsorption energy calculations predicted that Tyr coordination stabilizes the metal clusters, favouring the Ag_*n*_−Tyr complexes with an even number of silver atoms. For the Ag_*n*_ low-lying isomers, the general shape and the major transitions of the calculated time dependent-DFT (TD-DFT) absorption spectra align with those of previous reports measured in an argon environment. We use the analysis of non-covalent interactions to identify the specific interactions between each silver cluster and functional groups of Tyr. The TD-DFT absorption spectra for the Ag_*n*_−Tyr complexes showed that Tyr significantly modifies the optical properties of the coordinated silver clusters and affects the smaller systems to a greater extent. The optical absorption results of the bare Ag_*n*_ clusters and the Ag_*n*_−Tyr complexes are compared and discussed in detail.

## 1. Introduction

Noble metal clusters (NMCs) with a size around the Fermi wavelength of electrons (approx. 2−3 nm) display quantum confinement effects and properties such as photoluminescence, two-photon absorption and second and third harmonic generation [1]. As a result of these unique features, NMCs are increasingly gaining popularity in optics and catalysis [2–5]. However, NMCs are usually highly reactive, so it is standard to use capping agents to stabilize them [6]. Thus, the precise determination of the structure of coordinate complexes with ligands remains challenging despite advances in theoretical and experimental methods [7–9].

Previous theoretical works on bare silver clusters focused on predicting the structure and the corresponding optical absorption spectra [10]. For instance, Harb *et al.* [11] reported optical absorption measurements of small silver neutral clusters, Ag_*n*_ (*n* = 4−14) and obtained very similar spectra through time-dependent density functional theory (TD-DFT) computations. The accurate structural determination of the cluster is essential as it is directly linked to the optical properties. Similarly, experimental evidence indicates that each stoichiometry has virtually a unique absorption spectrum that may be used as the system fingerprint [12].

Also, silver clusters protected by proteins or peptides are promising candidates for biomedical applications, such as detecting heavy metal ions or as intracellular markers [13–16]. Despite this, most of the theoretical studies on protected metal clusters focus mainly on gold and comparatively fewer studies examine the interaction of silver clusters with ligands [17,18]. However, it should be noted that the incorporation of different types of ligands into a cluster may modify its optical absorption spectrum [19,20].

A practical and inexpensive stabilizing agent for NMCs is ovalbumin [21–23]. In this direction, Synch *et al.* [24] measured the fluorescence emitted by silver clusters synthesized in, among other types of proteins, ovalbumin. It is also essential to mention that tyrosine (Tyr) residues reduce the metallic ions in synthesizing silver and gold nanostructures using ovalbumin [25–27]. This evidence suggests that Tyr is one of the ovalbumin’s most representative amino acids as a stabilizing agent for silver nanostructures.

Herein, we present a study on the optical and structural properties of the complexes that form when a Tyr molecule protects silver clusters from 3 to 12 atoms. Recently, a related theoretical study was reported by Buglak & Kokonov [28], where small silver nanoclusters of up to eight atoms are coordinated with Tyr molecules. Unlike Buglak and Kokonov’s report, we compare the evolution of the absorption spectrum, calculated with a long-range corrected functional, for each Ag_*n*_−Tyr complex and the associated bare Ag_*n*_ cluster, finding an excellent agreement with the experimental evidence [11]. The study of these complexes illustrates one of the fundamental principles involved in the coupling of metallic nanoparticle–biomolecule systems at the atomic level and also provides a platform for developing nanomaterials with desired optical responses.

## 2. Methodology and computational details

We determine the putative global minima for the bare Ag_*n*_ (*n* = 3−12) clusters and the corresponding Ag_*n*_−Tyr complexes via the GLobal Optimization of MOlecular Systems (GLOMOS) [29]. GLOMOS is a code written in Python that, through different strategies, can explore the potential energy surface of atomic and molecular clusters by evaluating the energy through electronic structure codes. The global optimization strategy choice was a stochastic approach. GLOMOS generates trial structures with random atomic coordinates, ensuring all the atoms meet a proximity criterion and form a bonded assembly. To minimize the possibility of collapse into a funnel associated with a dominant morphology, GLOMOS generates trial structures with different morphological patterns. GLOMOS identifies and discriminates the equivalent configurations via the ultrafast shape-recognition algorithm with mass ponderation (USRAMP) proposed by Chen *et al.* [30]. A modified and open version of USRAMP is available in the Python package index (PyPI) repository [31].

The local optimization of the test structures Ag_*n*_ was done in two steps. All trial structures were fully optimized without symmetry constraints in the first stage using the B3PW91 hybrid functional [32] with the LanL2DZ basis set [33,34]. This functional-basis combination has delivered structural and electronic results in good agreement with the experimental data for small Ag naked clusters [35]. In the second stage, after the similarity discrimination process, the final structures were again fully optimized at the B3PW91 functional with the def2-TZVP basis set [36,37]. The highest multiplicities were not explored because the lowest spin state in small silver clusters is the ground state [38]. A harmonic frequency analysis was also performed to verify a stationary local minimum for all the optimized structures. The punctual group for each cluster was determined using the SYVA program [39] and the symmetry analyser module included in the Python Materials Genomics library [40].

On the other hand, the strategy to find the most energetically favourable Ag_*n*_−Tyr (*n* = 3−12) complexes consist of fixing the putative global minimum (GM) of each Ag_*n*_ cluster at the centre of a coordinate system trying different binding sites for Tyr. For each silver cluster, 30 × *n* possibilities were estimated by randomly changing the binding site and the spatial orientation of the molecule in its Euler angles. All the candidate Ag_*n*_−Tyr complexes thus built were fully optimized at the B3PW91/def2-TZVP level, including the Grimme D3 dispersion scheme [41]. All absorption spectra computations required the time-dependent Kohn–Sham formalism and related methods at the CAM-B3LYP [42] functional in combination with the def2-TZVP basis set [36,37]. Optical spectra data were managed with the graphical interface Gabedit [43].

A non-covalent interactions (NCIs) analysis at the B3PW91-D3/DZP level was performed for all the complexes to depict the coordination between the Ag_*n*_ clusters and Tyr. The mapping of the non-covalent regions was visualized using NCIPLOT [44,45] along with the VMD program [46]. The green and red colours for all the complexes represent stabilizing van der Waals and destabilizing steric interactions, respectively. All the DFT and TD-DFT computations were performed using the Gaussian 16 package [47].

## 3. Results

### 3.1. Structure and stability

#### 3.1.1. Ag_*n*_ (*n* = 3−12) clusters

**Figure 1.**
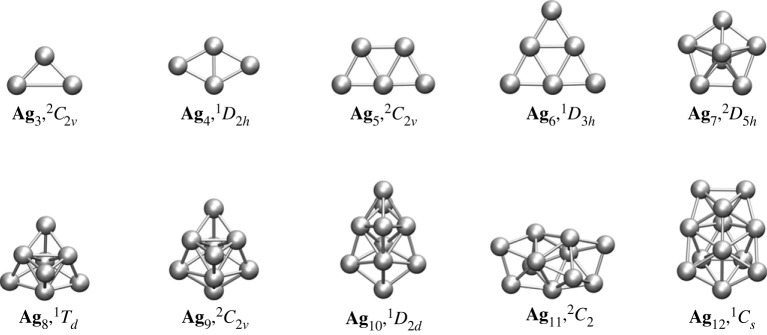
Global minima for the Ag_*n*_ clusters from *n* = 3−12 at the B3PW91/def2-TZVP level; each includes the spin multiplicity and associated symmetry group.

Figures 1 and 6 show the lowest-lying energy structures for the bare Ag_*n*_ (*n* = 3−12) clusters. The adopted geometry is planar for *n* = 3−6, whereas the clusters acquire three-dimensional shapes for *n* = 7−12. These results are in agreement with previous studies [11,28,38,48–56], except for those reported by Zhao *et al.* [57]. More specifically, the global minima of the Ag_*n*_ clusters with *n* = 3−7 is well-established in the literature: Ag_3_ is an isosceles triangle with *C*_2*v*_ symmetry [38,48,51,52,55–57], Ag_4_ has a rhombic *D*_2*h*_ symmetry [11,28,38,48,49,51,52,54–57], Ag_5_ has a planar trapezoidal *C*_2*v*_ form [11,28,38,48–53,55,56], Ag_6_ is a planar *D*_3*h*_ triangular arrangement [11,38,49–56] and Ag_7_ is a pentagonal bipyramidal *D*_5*h*_ non-planar structure [11,28,38,48–57]. However, there is still disagreement on the global minima for larger clusters. For the silver octamer, some authors suggested a tetra-capped tetrahedron *T*_*d*_ [11,28,38,49,50,56,58,59], while others proposed a *D*_2*d*_ distorted bicapped octahedron as the minimum energy structure [48,51,53–55,57]. Our search identified both geometries, finding the *T*_*d*_ structure 96.43 meV below the *D*_2*d*_ one. Moreover, this slight difference is reduced to 1.73 meV when the zero-point energy correction is added. The GM for the Ag_9_ cluster corresponds to a bicapped pentagonal bipyramid *C*_2*v*_ structure. Other groups have referred to this structure as the lowest-lying isomer [48,52,57,60]. On the other hand, some groups have reported a tricapped rectangular bipyramidal *C*_*s*_ structure (or tricapped-distorted octahedron) as the putative GM [11,38,51,53,55,56]. In this work, the *C*_*s*_ structure was ordered as the second lowest-lying energy and is 16.2 meV above the *C*_2*v*_ cluster. The most energetically favourable isomer for Ag_10_ is a pentagonal bipyramidal-shaped *D*_2*d*_ arrangement. Although most reports indicate such symmetry as the more stable form [11,51,52,55,60,61], a few isolated studies suggested the *D*_4*d*_ and *C*_*s*_ symmetries [38,57]. For Ag_11_, our method predicts two degenerated chiral *C*_2_ structures as the lowest-energy isomers. Other groups consider one of these chiral structures as the putative GM [11,51,52,60]. At the level of theory used, a *C*_1_ symmetry appears as the second-lowest energy isomer whose energy is 10.3 meV over the *C*_2_ structure. Nevertheless, other groups have proposed a *C*_2*v*_ structure as the low-lying energy isomer [38,62]; geometry 12.29 meV above our putative GM. The putative GM for the dodecamer Ag_12_ has the *C*_*s*_ symmetry. This structure matches the putative GM predicted by other groups with other distinct functionals and basis sets [11,38,51,52,60,61].

#### 3.1.2. Ag_*n*_−Tyr (*n* = 3−12) complexes

**Figure 2.**
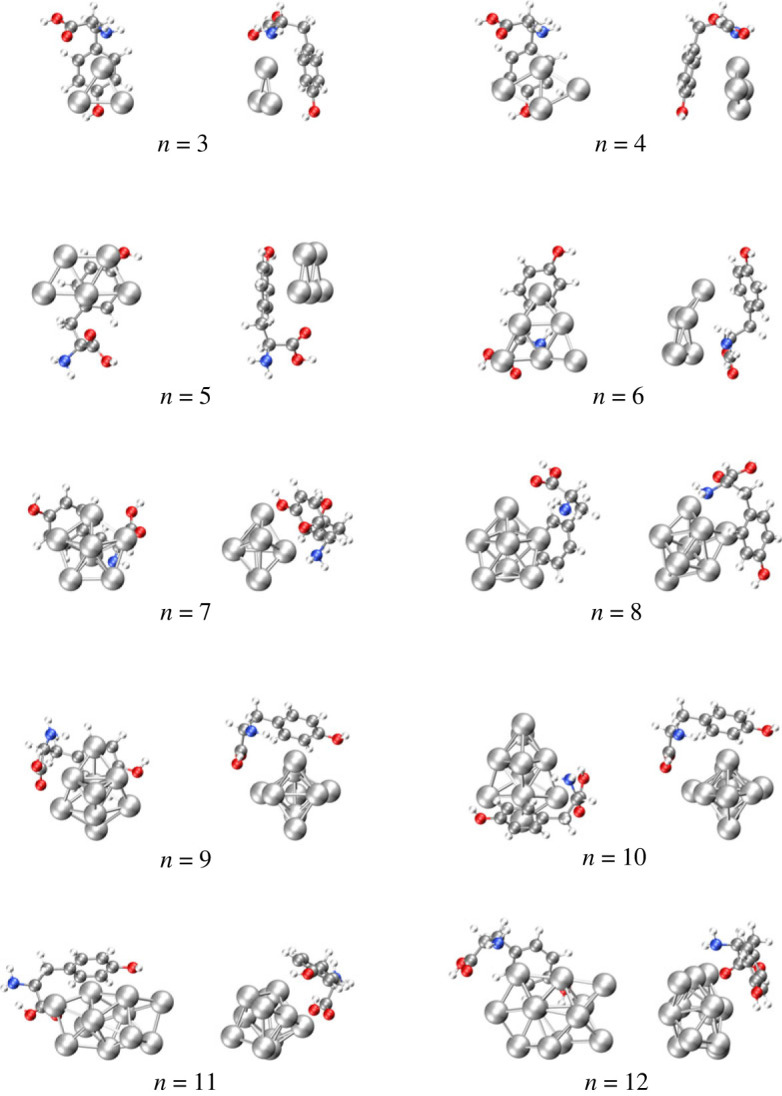
Minimum energy Ag_*n*_−Tyr complexes, from *n* = 3−12, at the B3PW91-D3/def2-TZVP level. Two perspectives are shown for each complex, one frontal and another lateral. The Ag atoms, the largest, are silver in colour, while the red, grey, blue and white spheres correspond to the O, C, N and H atoms.

Figure 2 shows the putative minimum energy structures for the Ag_*n*_−Tyr complexes, from *n* = 3 to 12 optimized at the B3PW91-D3/def2-TZVP level. Two perspectives for each complex are included to distinguish better the interaction between each silver cluster and Tyr. The Ag_*n*_ (*n* = 3−12) clusters coordinate with Tyr by interacting with the aromatic ring. In some cases, Tyr bends to maximize the interaction with the silver cluster. Each cluster coordinates with Tyr through strong interaction with one or more amino acid functional groups.

**Figure 3.**
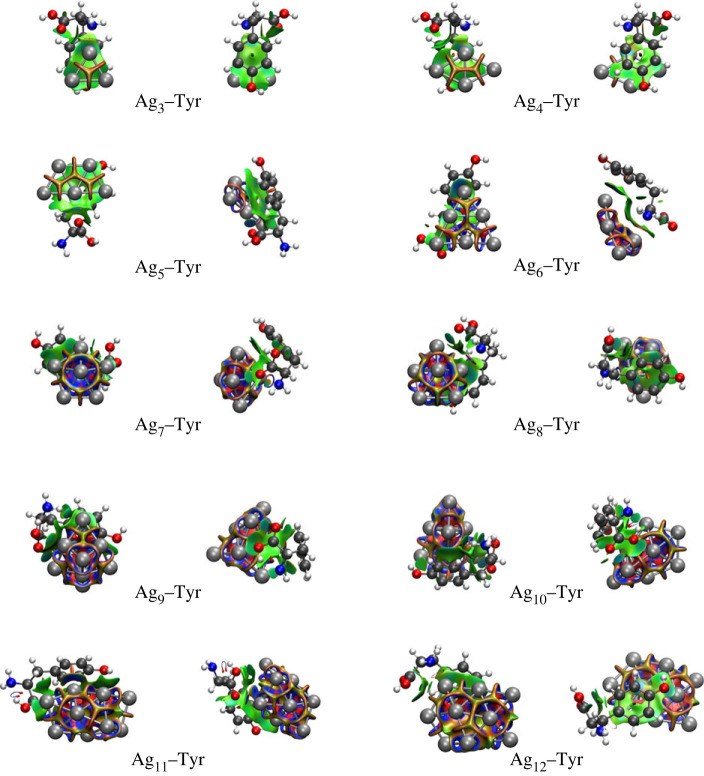
Ag_*n*_−Tyr complexes and their non-covalent interactions regions. The green surfaces correspond to weak NCIs, while the blue surfaces to strong and attractive interactions.

We use the NCI analysis to appreciate the interaction between each silver Ag_*n*_ cluster and Tyr. Figure 3 shows the NCI isosurfaces that characterize the interactions’ strength through the electronic density and curvature. The interaction’s colour code is as follows: blue corresponds to strong and attractive, green to weak, and red to strong and repulsive. For all complexes, the metallic bond between the silver atoms is the strongest interaction, while the cluster–Tyr coordination (see the green surface between the aromatic ring of the amino acid and the metallic clusters) is always weaker in comparison. On the other hand, the strong interaction regions (as blue colour sites) between an Ag atom in the cluster and Tyr are not the same for all the complexes. Also, not all complexes have the same number of interaction sites. For some complexes, Ag_*n*_−Tyr (*n* = 6, 8−11), the green surface includes one or more blue regions, indicating a strong interaction between an Ag atom of the cluster with a specific carbon atom in the aromatic ring. All complexes, except Ag_6_–Tyr, weakly interact with the hydroxyl group (OH) of phenol. Just Ag_7_–Tyr, Ag_10_–Tyr and Ag_12_–Tyr shows a strong interaction with the oxygen atom of the hydroxyl group. All the complexes that interact with the carboxyl group (−COOH): Ag_*n*_−Tyr (*n* = 9−11) do so with the hydroxyl group and the rest with a carbonyl group (C=O). On the other hand, the oxygen atom of carbonyl plays an essential role in the coordination between the amino acid and the metal cluster, this for Ag_3_−Tyr, Ag_5_−Tyr, Ag_7_−Tyr, Ag_8_−Tyr, Ag_9_−Tyr and Ag_11_−Tyr. Finally, two particular interactions are noteworthy, strong between Ag_6_−Tyr with the hydrogen atom bound to the *α*-carbon and weak between Ag_11_–Tyr and the *β*-carbon. For all the complexes, the most frequent Ag_*n*_−Tyr interaction site occurs at the oxygen atom in the carboxyl group C=O, in agreement with Buglak and Kokonov, who identify for Ag_*n*_−Tyr complexes with *n* ≤ 8 that the leading interaction site is the carboxylate and the second is the hydroxyl [28].

**Figure 4.**
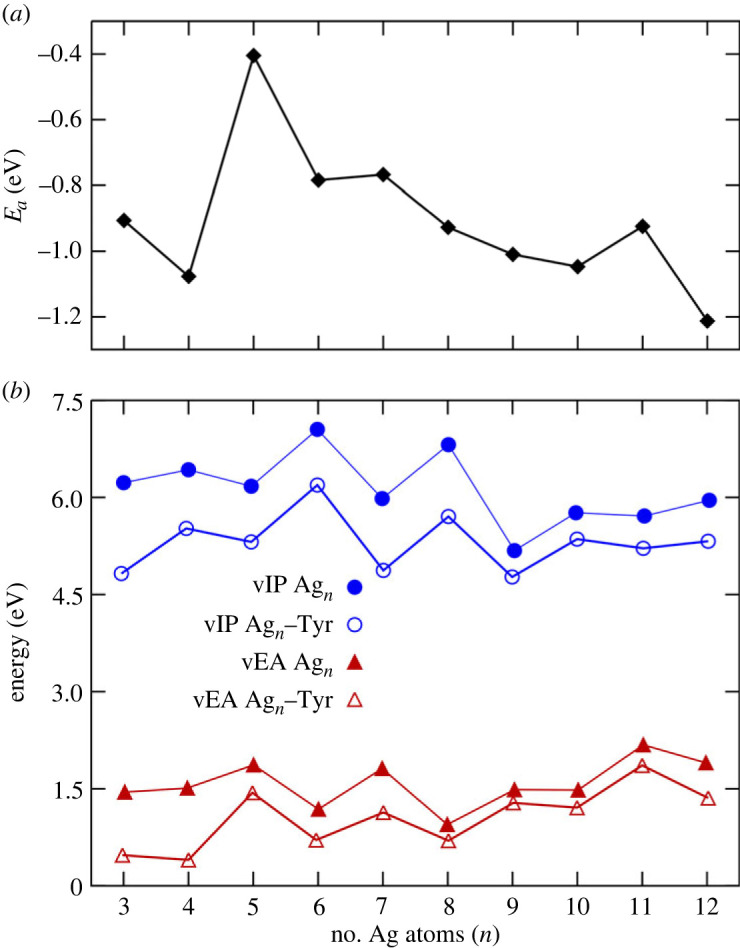
(*a*) Adsorption energy for each Ag_*n*_−Tyr complex. (*b*) Comparison of the vIP (circles) and vEA (triangles) for the Ag_*n*_ clusters (filled symbols) and Ag_*n*_−Tyr complexes (open symbols).

We compute the adsorption energy (*E*_*a*_), vertical ionization potential (vIP) and vertical electron affinity (vEA) to analyse the stability of the complexes. It is essential to mention that previous work has shown silver neutral clusters have thermodynamic and chemical stability, calculating parameters such as the binding energy [63]. The binding energy calculates the energy gained during the cluster formation from its atoms [64,65]. For the Ag_*n*_−Tyr complexes, we define the adsorption energy as the decreasing energy between its components: the Ag_*n*_ cluster and a tyrosine molecule, in the form:

3.1 $$E_{a}=E[{\mathrm{Ag}}_{n}-\mathrm{Tyr}]-(E[{\mathrm{Ag}}_{n}]+E[\mathrm{Tyr}]),$$

where $E[{\mathrm{Ag}}_{n}-\mathrm{Tyr}]$, *E*[Ag_*n*_] and *E*[Tyr] are the total energies for the complex, the bare Ag_*n*_ cluster and the isolated Tyr, respectively. The adsorption energy is negative if the complex is more stable than its separate components. Thus, a more negative *E*_*a*_ value denotes a more strongly bonded complex. Figure 4*a* shows the adsorption energy as a function of the cluster size.

Adsorption energy results suggest that a cluster with an even number of silver atoms, coordinated with a Tyr, achieves relatively higher stability than a neighbouring complex with an odd number of silver atoms could reach. Figure 4*a* shows that complexes with relatively low *E*_*a*_ values, the most favourable for formation, always are coordinated with a cluster with an even number of silver atoms. Thus, the Ag_4_–Tyr complex is more favourable than their neighbouring Ag_3_–Tyr and Ag_5_–Tyr complexes. The same occurs for the Ag_6_–Tyr and Ag_10_–Tyr complexes concerning their adjacent neighbours. By contrast, the highest *E*_*a*_ values correspond to the complexes where the silver clusters are less likely to coordinate with a Tyr. An odd number of atoms forms most silver clusters in this category. This lower stability than its neighbouring odd-size clusters can be attributed to electronic shell closing effects [66]. Also, the adsorption energy trend suggests that the coordination with Tyr will be further favoured for even-size clusters with more than 12 silver atoms.

The vIP and vEA estimate the relative stability in the complexes by increasing the number of silver atoms. For a neutral system, the vIP is the energy difference between the cationic and neutral states in the equilibrium geometry. If the energy of the anionic state is used instead of the corresponding cation, then the energy difference is the vEA. Thus, the vIP and vEA for the bare Ag_*n*_ clusters and the Ag_*n*_−Tyr complexes are defined as follows:

3.2 $$\begin{array}{rl} & \text{vIP}[{\mathrm{Ag}}_{n}]=E[{\mathrm{Ag}}_{n}^{+}]-E[{\mathrm{Ag}}_{n}],\\ & \text{vIP}[{\mathrm{Ag}}_{n}-\mathrm{Tyr}]=E[{\mathrm{Ag}}_{n}-{\mathrm{Tyr}}^{+}]-E[{\mathrm{Ag}}_{n}-\mathrm{Tyr}],\\ & \text{vEA}[{\mathrm{Ag}}_{n}]=E[{\mathrm{Ag}}_{n}]-E[{\mathrm{Ag}}_{n}^{-}]\\ \;\mathrm{and}\; & \text{vEA}[{\mathrm{Ag}}_{n}-\mathrm{Tyr}]=E[{\mathrm{Ag}}_{n}-\mathrm{Tyr}]-E[{\mathrm{Ag}}_{n}-{\mathrm{Tyr}}^{-}]. \end{array}\}$$

Figure 4*b* shows the vIP and vEA evolution for the Ag_*n*_ and Ag_*n*_–Tyr complexes as a function of the number of silver atoms *n*. The vIP behaviour is similar for the clusters and the complex systems, i.e. the maximum values of vIP occur at n=6 and 8 and the minimum values at n=7 and 9. By contrast, for the other complexes in the series, the vIP is relatively steady. Thus equations (3.1) and (3.2) have a distinct order of factors to obtain a positive value. Furthermore, from vEA results, neutral Ag_*n*_ clusters and Ag_*n*_–Tyr complexes are less stable than their anionic counterparts. Table 1 summarizes the vIP results, making a comparison with experimental and theoretical results for bare Ag_*n*_ clusters [55,67]. The experimental values are generally very similar, with disagreements ranging from 0.6% to 15.3%. These differences could be associated with this cluster’s current geometry of the GM. Notably, the vEA values of *n* = 8 and 10 are almost identical for both cases, cluster and complex, in correlation with the relatively high stability of the bare silver clusters with an even number of atoms.

**Table 1.** Comparison of the vertical ionization potential (vIP) for the Ag_*n*_ and Ag_*n*_–Tyr, from *n* = 3–12. (All values are in eV.)

*n*	Ag_*n*_	Ag_*n*_	Ag_*n*_	Ag_*n*_–Tyr
	(this work)	[55]	exp. [67]	(this work)
3	6.24	5.67	6.20	4.85
4	6.45	6.54	6.65	5.55
5	6.18	6.33	6.35	5.37
6	7.06	7.22	7.15	6.24
7	6.00	6.36	6.40	4.95
8	6.86	6.44	7.10	5.76
9	5.20	6.53	6.00	4.81
10	5.84	6.58	6.25	5.38
11	5.76	6.66	6.30	5.25
12	6.01	7.01	6.50	5.38

#### 3.1.3. Ultraviolet-visible absorption spectra

**Figure 5.**
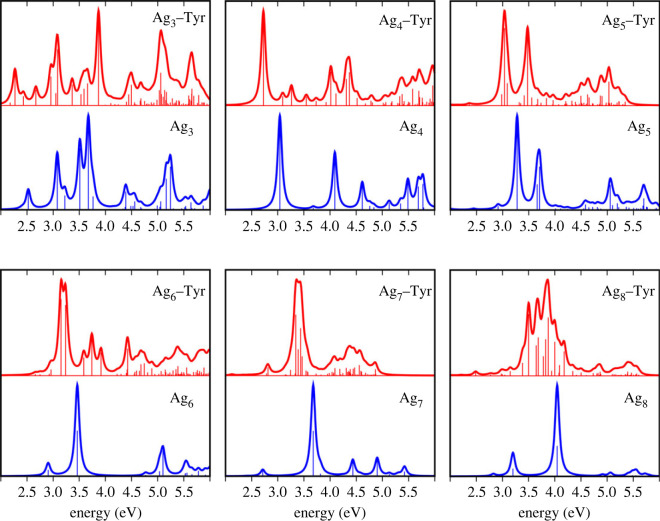
UV–Vis absorption spectra of the Ag_*n*_ clusters (blue line) and the Ag_*n*_–Tyr complexes (red line) for *n* = 3–8. Each spectrum includes the calculated oscillator strengths as vertical sticks in arbitrary units.

**Figure 6.**
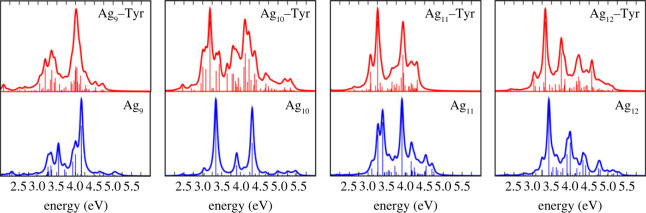
UV–Vis absorption spectra of the Ag_*n*_ clusters (blue line) and the Ag_*n*_–Tyr complexes (red line) for *n* = 9–12. Each spectrum includes the calculated oscillator strengths as vertical sticks in arbitrary units.

Table 2 compares the significant transitions from ultraviolet-visible (UV–Vis) computations with the experimental results of small silver clusters in an argon matrix at 28 K [11,56]. Figures 5 and 6 show the UV–Vis absorption spectra of the Ag_*n*_ clusters and Ag_*n*_–Tyr complexes. For small Ag_*n*_ clusters (*n* = 3–8), discrete peaks are shown in the range from 2.5 to 5.0 eV, while for larger clusters, the absorption curve shows the formation of bands, consistent with the results of Harb *et al.* [11]. The 2.0–6.0 eV range choice agrees with TD-DFT calculations and the experimental results on larger neutral Ag_*n*_ clusters that centre the main absorption band at 4.2 eV [68].

**Table 2.** Main peaks comparison between calculated and experimental absorption spectra on small naked Ag_*n*_ clusters and Ag_*n*_–Tyr complexes. (All units are in eV. The values for *n* = 10 are not included owing to the experimental difficulties associated with the spectrum measurement (see the text).)

	Ag_*n*_		Ag_*n*_–Tyr
*n*	(this work)	exp. [11,56]	(this work)
3	3.09, 3.66, 5.21	3.71, 3.51, 2.53, 3.07	3.86, 5.06, 3.07, 5.65
4	3.04, 4.09, 5.73	3.07, 3.21, 4.50	2.72, 4.34, 5.95, 5.72, 4.01
5	3.27, 3.69	3.75, 3.25	3.04, 3.47
6	3.46, 5.08	3.63, 4.15, 4.92	3.14, 3.23, 3.74, 4.42
7	3.67, 4.90, 4.43	3.60, 3.75, 2.81, 4.50	3.39, 4.42
8	4.04, 3.20	3.90, 3.82, 3.57, 3.20	3.85, 3.68, 3.51
9	4.16, 4.00, 3.54, 3.32	4.14, 3.97, 3.80, 3.65, 2.84	4.01, 3.36, 3.20
10	3.35, 4.31, 3.90	3.70, 3.95, 4.35	3.18, 4.13, 3.82, 3.41
11	3.90, 3.37, 3.27, 4.15	3.67, 4.31, 3.06	3.25, 3.92, 4.14, 3.07, 4.28
12	3.43, 3.96, 4.34	3.42, 3.91, 4.38	3.33, 3.76, 4.21, 4.56

For the Ag_3_ cluster, the central peak is 3.66 eV, and the second most intense signal is 3.09 eV. The region above 4.0 eV shows relevant peaks, where the third most intense signal is at 5.21 eV. For Ag_3_–Tyr, the primary signal is at 3.86 eV, representing a blue shift of 0.77 eV concerning the Ag_3_ spectrum. For the coordinate complex, the second most intense signal is at 5.06 eV.

In the interval between 2.0 and 6.0 eV the Ag_4_–Tyr absorption spectrum shows more peaks than the Ag_4_ reference. The central absorption peak is at 3.04 eV for Ag_4_ and at 2.72 eV for Ag_4_–Tyr, indicating that the coordination with Tyr induced a small red-shift of 0.32 eV.

In the Ag_5_ spectrum, two main absorption signals in descending order are at 3.27 and 3.69. Experimental measurements in Ar at 10 K revealed significant transitions at 3.25 and 3.75 eV [69], confirming that the spectrum is not very sensitive to the inert gas environment. In descending order, the principal peaks in the Ag_5_–Tyr spectrum are at 3.04 and 3.47 eV, representing an approximately 0.23 eV red rigid shift concerning the Ag_5_ spectrum.

The most intense peak absorption for the Ag_6_ cluster is at 3.46 eV, with a secondary signal at 5.08 eV. The experimental spectrum for the silver hexamer in Ar shows two double peaks at 3.63 and 4.15 eV and two broad and less intense peaks at 4.92 and 5.10 eV [11]. The spectrum in the current work reveals the precise position of two of the four prominent peaks in the experiment, even matching the order of their intensities. In agreement with Harb *et al.* [11], the missing secondary peaks are by the contribution of the next low-lying energy isomer. This is explained because, experimentally, the source of the cluster could produce isomers lying very close in energy that coexists in the molecular beam. In the Ag_6_–Tyr spectrum, the leading four signals are found in the range between 3.14 and 4.42 eV, where the principal peak (at 3.14 eV) represents a red-shift of 0.32 eV for the Ag_6_ spectrum.

The two more prominent peaks in the Ag_7_ spectrum are at 3.67 and 4.90 eV, with a third low-intensity peak at 4.43 eV. The experimental spectrum shows broadband with two maxima at 3.6 and 3.75 eV and small transitions around 4.5 and 5.0 eV [11]. The comparison with the experimental measurement is satisfactory, newly attributed to the Ag_7_ high relative stability. The Ag_7_–Tyr spectrum showed a red-shift at the most intense absorption peak. The highest absorption for the complex is at 3.39 eV instead of 3.67 eV.

Optical absorption calculation for Ag_8_ brought two central values, from highest to lowest intensity at 4.04 and 3.20 eV. Our TD-DFT spectrum closely resembles the UV–Vis measured by Harb *et al*. [11], showing transitions at 3.90 and 3.20 eV corresponding to the GM and peaks at 3.57 and 3.82 eV associated with the second lowest-energy isomer. By contrast, the Ag_8_–Tyr spectrum shows more peaks with the highest intensity at 3.85 eV, which indicates a slight red shift of 0.19 eV. Other notable signals for the complex are at 3.68 and 3.51 eV.

Figure 6 shows the spectra for the largest systems in the series (*n* = 9–12). The absorption spectrum of Ag_9_ indicates a high-intensity signal at 4.16 eV and the following in intensity at 4.00 eV. Also, a third-order peak appears at 3.54 eV and a fourth-order signal at 3.32 eV. Because of the complexity of this spectrum, it can be summarized that the Ag_9_ cluster with *C*_*s*_ symmetry has its most prominent peaks between 3.20 and 4.20 eV. The experimental spectrum of Ag_9_ in Ar at 28 K is a broader composition of distinct and narrow peaks at 2.84, 3.65, 3.80, 3.97, 4.14 and 4.95 eV [11]. Harb and his collaborators conclude that the experimental spectra can be understood as a sum of contributions of the first three isomers in the matrix. Interestingly, our TD-DFT spectra for the GM structure already describe the main shape of the experimental absorption spectrum, unlike those reported by Harb *et al*.

From an experimental viewpoint Ag_10_ was the most challenging system, showing three broad peaks in its spectrum at 3.70, 3.95 and 4.35 eV [11]. In comparison, our TD-DFT absorption spectra show the most prominent peaks at 3.35, 3.90 and 4.31 eV. Being optimistic, we can think that such peaks correspond to each other. Nevertheless, Harb and co-workers reported that none of their calculated spectra or the sum of them reproduced the experimental spectra. Therefore, they suggest that discrepancies could be owing to the low signal-to-noise ratio of the experimental spectra. Our best correlation with the experiment could be attributed to including the long-range corrected CAM-B3LYP functional; this is because our test calculations for the Ag_10_ adsorption spectrum via the B3PW91 functional practically reproduces the Harb *et al.* [11] theoretical result. The Ag_10_–Tyr and Ag_10_ absorption spectra showed some similarities, as the location of the central peak at 3.28 eV. Essentially, two intense peaks form the Ag_10_ spectrum, and the rest are low-intensity signals. By contrast, the Ag_10_–Tyr spectrum shows a series of medium and low-intensity signals between 2.89 and 4.24 eV, with shallow signals out of this range. The appearance of these signals of medium, low and very low intensity may be the result of Tyr coordination.

In order of intensity, the UV–Vis absorption spectrum of Ag_11_ shows four prominent peaks at 3.90, 3.37, 3.27 and 4.15 eV. The experimental Ag_11_ spectrum in Ar at 28 K shows three relevant peaks at 4.31, 3.67 and 3.06 eV [11]. The measurements show different relative intensities, indicating that this system is sensitive to the environment [11,70,71]. Harb and co-workers again conclude that the experimental spectra might not be related to a specific isomer. Besides, previously detailed calculations in the silver undecamer suggest that the lowest-lying *C*_1_ isomer yields the best approximation to the experimental spectra [72]. Unlike these authors, we have found a good correlation with the experimental data, considering only the GM structure. Again, we attribute our better correlation with the experiment to including the long-range corrected CAM-B3LYP functional. The Ag_11_–Tyr spectrum shows two remarkable peaks: the highest at 3.25 and the second at 3.92 eV. Similar to previous coordinate complexes, the central peak of the Ag_11_–Tyr spectrum is red-shifted in 0.42 eV compared with the Ag_11_ reference.

For the largest cluster in the series, Ag_12_, the signal at 3.43 eV is the most intense, with the next prominent peaks at 3.96, 4.34 and 4.77 eV. The Ag_12_ experimental spectrum shows two prominent broad peaks at 3.42 and 3.91 eV and one less intense at 4.38 eV [11]. Our TD-DFT spectrum agrees very well with the general shape of the experimental measurements. Thus, the GM *C*_*s*_ symmetry represents the main contribution to the experimental absorption spectra. For the Ag_12_–Tyr complex, the maximum intensity absorption is 3.33 eV, and left to this point is the second most intense absorption at 3.76 eV. The rest of the signals in the spectrum are of medium or low intensity, contrary to the spectra of bare dodecamer, where there are three peaks of considerable intensity.

## 4. Conclusion

In this work, we explored the potential energy surface for neutral Ag_*n*_ clusters and their corresponding complex with a Tyr molecule, Ag_*n*_–Tyr (*n* = 3–12). The putative global minima structures for the Ag_*n*_ (*n* = 3–12) clusters agree well with previous reports on the structure and related descriptors, such as the adsorption energy, vertical ionization potential and electronic affinities.

For the range from n=3 to 12, vertical electron affinity calculations suggest that the Ag_*n*_–Tyr complexes in the neutral state are energetically more favourable compared to the corresponding Ag_*n*_ neutral clusters, where this difference is more evident when *n* is an even number. The NCI analysis shows that the strong interaction sites between silver atoms and Tyr vary for each complex. The most frequent Tyr interaction site for all coordinated complexes occurs at the carboxyl group’s oxygen atom C=O, usually as a weak interaction between the metallic cluster and the phenol aromatic ring.

Our TD-DFT UV–Vis spectra computed for each Ag_*n*_ low-lying energy isomer at the CAM-B3LYP/def2-TZVP level agrees with the general shape and significant transitions of the reported absorption spectra measured in Ar [11]. The inclusion of the long-range corrected CAM-B3LYP functional is decisive in obtaining a better agreement with the experimental adsorption spectrum for the Ag_10_, Ag_11_ and Ag_12_ clusters, considering that previous TD-DFT spectra for these clusters at the BP86/LANL2DZ level are the ones that most disagree with the experimental UV–Vis measurements [11]. Our result also confirms that although the inclusion of other higher-energy isomers is essential, the main characteristics of the absorption spectra can be described by considering the low-energy isomer of each system. Our results suggest that the coordination of the Ag_*n*_ clusters with a Tyr molecule promotes changes in the absorption spectra from the ultraviolet (UV-C, 4.5 eV) in the bare metal clusters to the near UV (3.10–4.13 eV) for the Ag_*n*_–Tyr complexes.

## Data Availability

Data are available from the Dryad Digital Repository: https://doi.org/10.5061/dryad.cfxpnvxbk [73].
